# Fenofibrate Recognition and G_q_ Protein Coupling Mechanisms of the Human Cannabinoid Receptor CB1

**DOI:** 10.1002/advs.202306311

**Published:** 2024-01-31

**Authors:** Tianxin Wang, Wenqin Tang, Ziyi Zhao, Ran Zhao, Zhenyu Lv, Xuzhen Guo, Quanchang Gu, Boxiang Liu, Haoyu Lv, Jiayan Chen, Kaiquan Zhang, Fahui Li, Jiangyun Wang

**Affiliations:** ^1^ CAS Key Laboratory of Quantitative Engineering Biology Institute of Synthetic Biology Shenzhen Institute of Advanced Technology Chinese Academy of Sciences Shenzhen 518055 China; ^2^ iHuman Institute ShanghaiTech University 393 Middle Huaxia Road Pudong Shanghai 201210 China; ^3^ Institute of Biophysics Chinese Academy of Sciences 15 Datun Road Chaoyang District Beijing 100101 China; ^4^ School of Life Sciences University of Chinese Academy of Sciences Beijing 100049 China; ^5^ Key Laboratory of Biomacromolecules Chinese Academy of Sciences Beijing 100101 China

**Keywords:** cryo‐electron microscopy, fenofibrate, G_q_ coupling, human cannabinoid receptor 1, ligand recognition

## Abstract

The G‐protein‐coupled human cannabinoid receptor 1 (CB1) is a promising therapeutic target for pain management, inflammation, obesity, and substance abuse disorders. The structures of CB1‐G_i_ complexes in synthetic agonist‐bound forms have been resolved to date. However, the commercial drug recognition and G_q_ coupling mechanisms of CB1 remain elusive. Herein, the cryo‐electron microscopy (cryo‐EM) structure of CB1‐G_q_ complex, in fenofibrate‐bound form, at near‐atomic resolution, is reported. The structure elucidates the delicate mechanisms of the precise fenofibrate recognition and G_q_ protein coupling by CB1 and will facilitate future drug discovery and design.

## Graphical abstract

The cryo–EM structure of CB1‐G_q_ complex, in fenofibrate‐bound form, is resolved to elucidate the ligand selectivity of fenofibrate and G_q_ coupling mechanism to CB1, providing a structural understanding of ligand recognition and G_q_ coupling mechanisms of CB1.

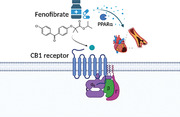

## 1 Introduction

CB1 is the principal target of Δ^9^‐tetrahydrocannabinol (THC), a psychoactive chemical from Cannabis sativa L. with a wide range of therapeutic applications and recreational uses for more than 5000 years.^[^ ^1^, ^2^ ^]^ CB1 also mediates diverse pathophysiological effects of endocannabinoids like anandamide (AEA) and 2‐arachidonoyl glycerol (2‐AG),^[^ ^3^, ^4^ ^]^ and numerous synthetic cannabinoids^[^ ^5^ ^]^ by coupling to G proteins of the G_i/o_ and G_q_ classes.^[^ ^6^, ^7^ ^]^ As the most abundant receptor in the mammalian brain,^[^ ^1^ ^]^ CB1 mainly distributes in the central nervous system and exerts regulatory influence over cognitive functions, memory, and motor control by modulating neurotransmitter release, which is regarded as one important target of mental illness such as depression and anorexia nervosa.^[^ ^8^ ^]^

Fenofibrate, ranked among the world's top 200 drugs by sales, is widely prescribed for treating primary hypercholesterolemia, mixed dyslipidemia, and hypertriglyceridemia. Acting through peroxisome proliferation‐activated receptor (PPAR), fenofibrate effectively lowers blood cholesterol and triglyceride levels.^[^ ^9^ ^]^ Beyond its lipid‐lowering effects, fenofibrate has demonstrated central nervous system pharmacological impacts, including antidepressant‐like effects in mice^[^ ^10^ ^]^ and neuroprotection against Parkinson's disease. Notably, these effects may be mediated through the CB1 signaling pathway, as Fenofibrate has been identified as a partial agonist of CB1.^[^ ^11^, ^12^ ^]^

While various active and inactive CB1 structures in synthetic cannabinoid‐bound forms have been resolved to clarify the ligand selectivity,^[^ ^13^, ^14^, ^15^, ^16^, ^17^, ^18^, ^19^ ^]^ the commercial drug recognition and G_q_ coupling mechanisms of CB1 remain obscure. The lack of experimental structure of fenofibrate‐bound CB1‐G_q_ complex has hindered understanding of the pathophysiological function underlying CB1 signaling. Herein, we report the cryo‐EM structure of CB1‐G_q_ complex, in fenofibrate‐bound form, to unravel the ligand selectivity of fenofibrate for CB1 and shed light on the G_q_ coupling mechanism, providing a structural understanding of ligand recognition and G_q_ coupling mechanisms of CB1.

## 2 Results and Discussion

### 2.1 Overall Cryo‐EM Structure of the Fenofibrate‐Bound CB1 Complex

**Figure 1:**
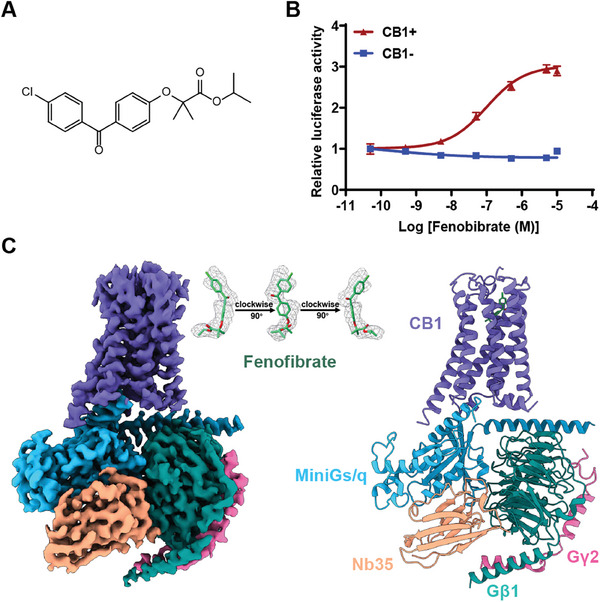
Overall cryo‐EM structure of the fenofibrate‐boundCB1‐miniG_s/q_β_1_γ_2_‐Nb35 complex. A) Chemical structure of fenofibrate. B) The calcium‐induced luciferase accumulation assays for fenofibrate‐induced CB1 activation. Data are shown as the mean ± s.e.m. from three independent measurements. C) Cryo‐EM density map and model of the fenofibrate‐bound CB1‐miniG_s/q_β_1_γ_2_‐Nb35 complex.

In our study, the calcium‐induced luciferase accumulation assays indicated that fenofibrate could activate CB1 through G_q_ signaling pathway (**Figure** **1**A,B). To obtain a stable and homogeneous cyro‐EM sample, we introduced the miniG_s/q_ protein and employed the NanoBiT strategy to stabilize the fenofibrate‐bound CB1‐G_q_ complex.^[^ ^20^, ^21^ ^]^ The CB1, miniG_s/q_ protein and β_1_γ_2_ were expressed in HEK293F, *Escherichia Coli* and sf9 cells, respectively. These proteins were purified and assembled in vitro with the addition of fenofibrate and Nb35,^[^ ^22^ ^]^ which stabilizes the nucleotide‐free complex by bridging the miniG_s/q_ and Gβ_1_γ_2_ subunits. All of these components were identified through SDS‐PAGE analysis (Figure S1, Supporting Information). The structure was determined using single‐particle cryo‐EM, yielding an overall resolution of 2.9 Å (Figure 1C; Figure S2 and Table S1, Supporting Information). The high‐quality cryo‐EM map enabled accurate modeling of the 7TM elements of the CB1 receptor, the miniG_s/q_β_1_γ_2_ heterotrimer, and Nb35. Notably, the side chains of most residues are well defined in all components (Figure S3, Supporting Information). Furthermore, fenofibrate was unequivocally identified within the orthosteric pocket of CB1 (Figure 1C).

### 2.2 Fenofibrate Recognition by CB1

**Figure 2:**
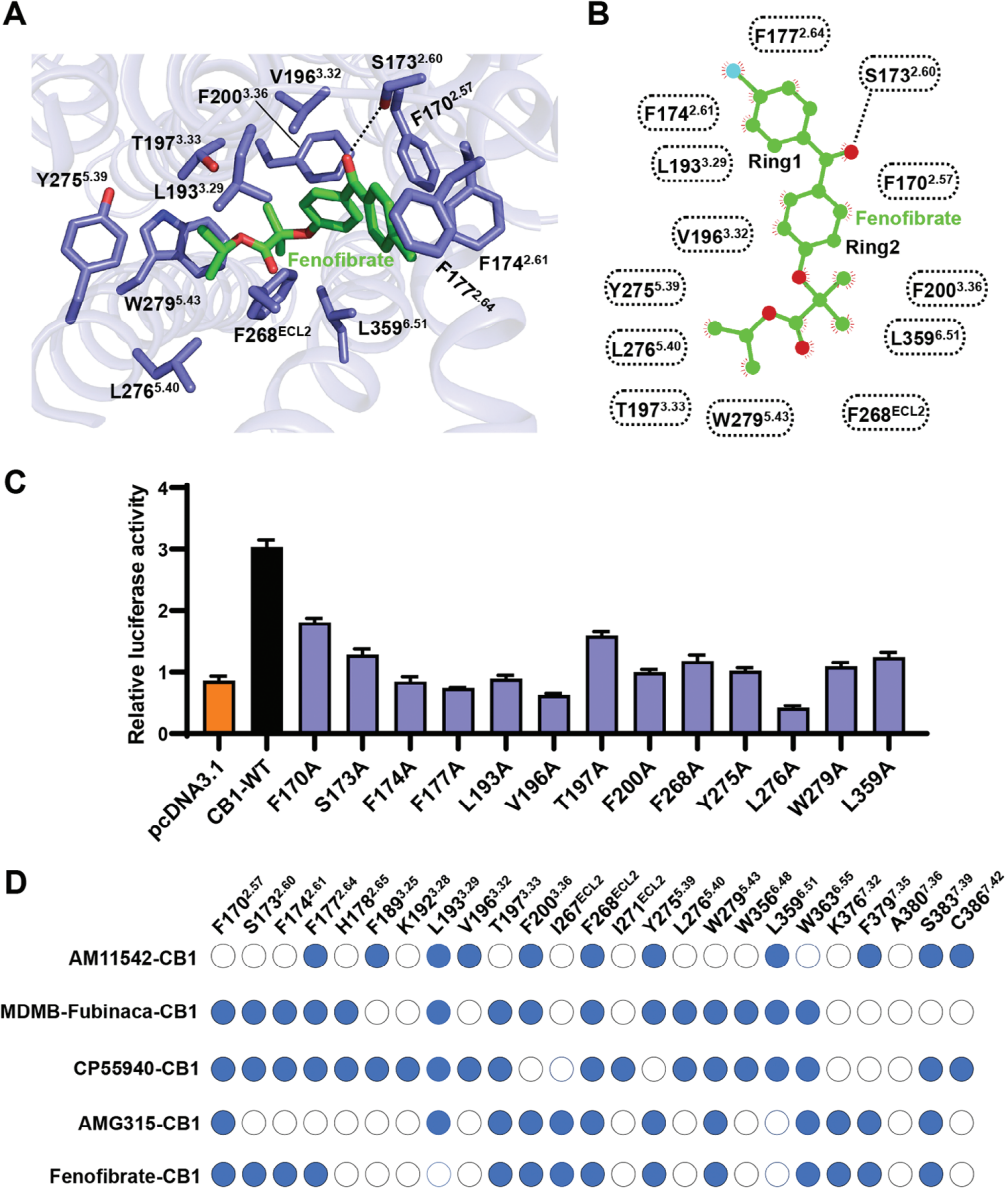
Architecture of the fenofibrate binding site in CB1. A) Interactions between fenofibrate (green) with CB1 (slate blue). Polar interactions are shown as dashed lines. B) 2D representation of contacts between CB1 and ligand fenofibrate shown in A. C) Mutational effects of CB1's orthosteric pocket on fenofibrate (5 µm) induced increased calcium activities. Data are shown as the mean ± s.e.m. from three independent measurements. D) Interaction differences in the orthosteric pocket of the CB1 bound by fenofibrate and other agonists (PDB code: 5XRA, 6N4B, 6KQI, and 8GHV).

In elucidating the interaction details between CB1 and fenofibrate, we observed substantial structural rearrangements within the orthosteric pocket of the fenofibrate‐bound CB1 complex. While the agonist fenofibrate adopted an L‐shape conformation closely resembling that seen in other agonist‐bound CB1 structures (Figure S4, Supporting Information), notable distinctions emerged within the orthosteric pocket. The interactions between fenofibrate and CB1 are mainly hydrophobic and aromatic, consisting of residues from extracellular loop 2 (ECL2), transmembrane helices (TM) 2, 3, 5, and 6 (**Figure** **2**A). Specifically, the benzene ring (Ring1) of fenofibrate engaged in π–π interaction with the residue F174^2.61^ (superscripts indicate Ballesteros‐Weinstein numbering for GPCRs^[^ ^23^ ^]^). Additionally, fenofibrate was involved in extensive hydrophobic interactions with the residues F170^2.57^, F177^2.64^, L193^3.29^, T197^3.33^, F200^3.36^, Y275^5.39^, L276^5.40^, W279^5.43^, L359^6.51^, and F268^ECL2^ (Figure 2A,B). These interactions were further confirmed by mutagenesis analysis (Figure 2C). Of note, the carbonyl group at the C12 position of fenofibrate forms an additional hydrogen bond with the residue S173^2.60^ (Figure 2A), which was also observed in the CP55940 binding pocket.^[^ ^24^ ^]^ Furthermore, the alignment map of CB's agonists binding pocket indicated that fenofibrate adopted a different barcode for CB1 interaction compared to other agonists‐bound CB1 structures (Figure 2D), perhaps accounting for the fenofibrate recognition specificity of CB1.

**Figure 3:**
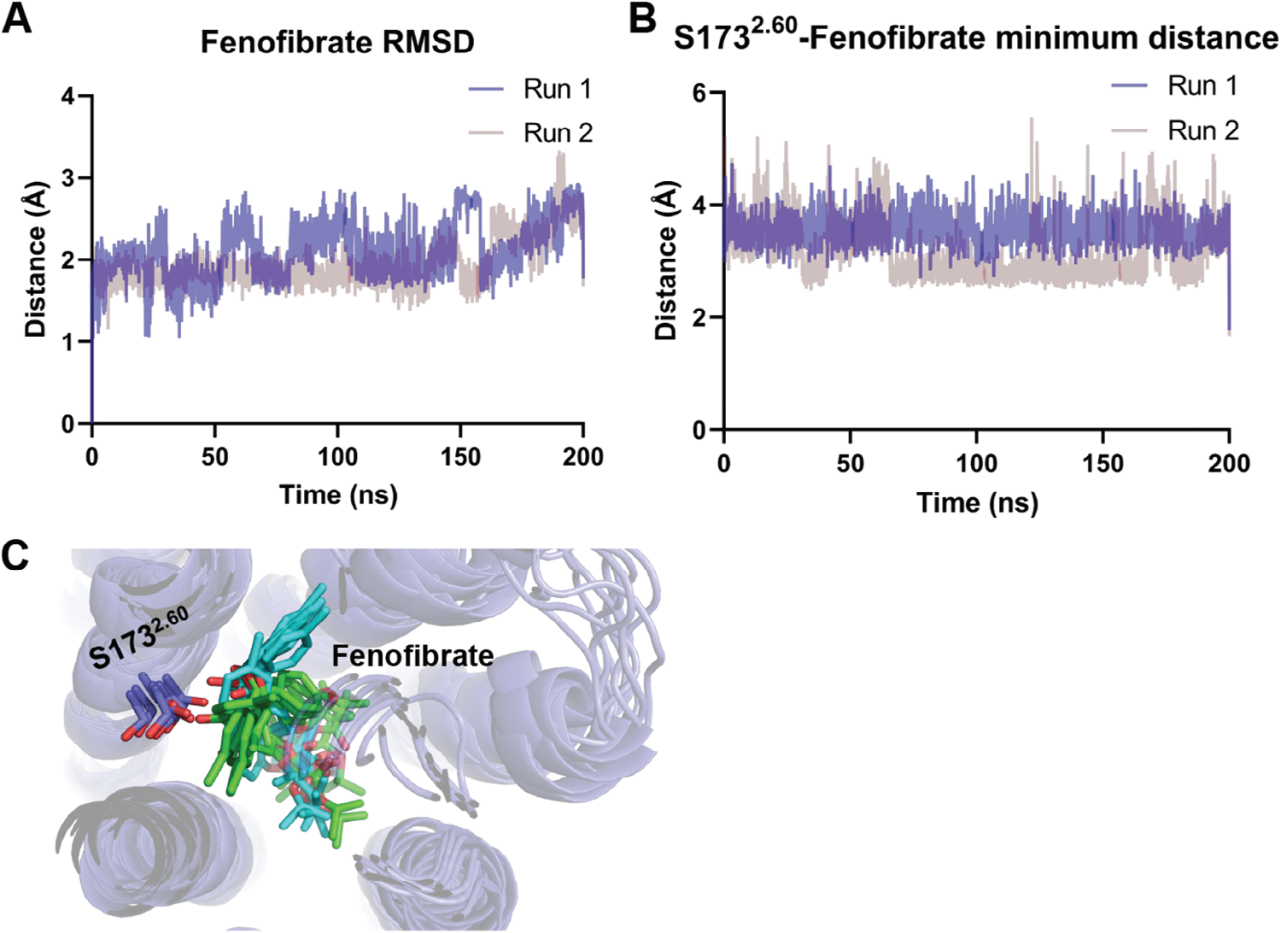
MD simulations of CB1 with fenofibrate. A) RMSD over times of fenofibrate in fenofibrate‐bound CB1 system. B) The minimum distances of heavy atoms between fenofibrate and the side chain of S173^2.60^. C) Snapshots alignments from evenly sampling of 40 ns interval of two parallel MD simulations (Run 1 with green ligand; Run 2 with cyan ligand).

To further identify the stability of fenofibrate in the orthosteric pocket, the molecular dynamics (MD) simulation was carried out to elaborate the interaction between fenofibrate and CB1. During two parallel 200 ns canonical NPT ensemble simulations, we found that the fenofibrate remains a relatively constrained conformation but may exist as a flexible binding mode, as reasoned from root mean square deviations (RMSD) and quite diverse contact frequency of fenofibrate with surrounding residues during simulation (**Figure** **3**A; Figure S5, Supporting Information). Two simulations brought fenofibrate into two different “Ring 1” orientations (Figure 3C). As expected, the residue S173^2.60^ kept quite close contact as the same in an initial model in two simulations (Figure 3B,C). Along with prevalent hydrophobic and van der Waals interactions, fenofibrate was caged in the orthosteric‐binding pockets.

### 2.3 The G_q_ Coupling Mechanism of CB1

**Figure 4:**
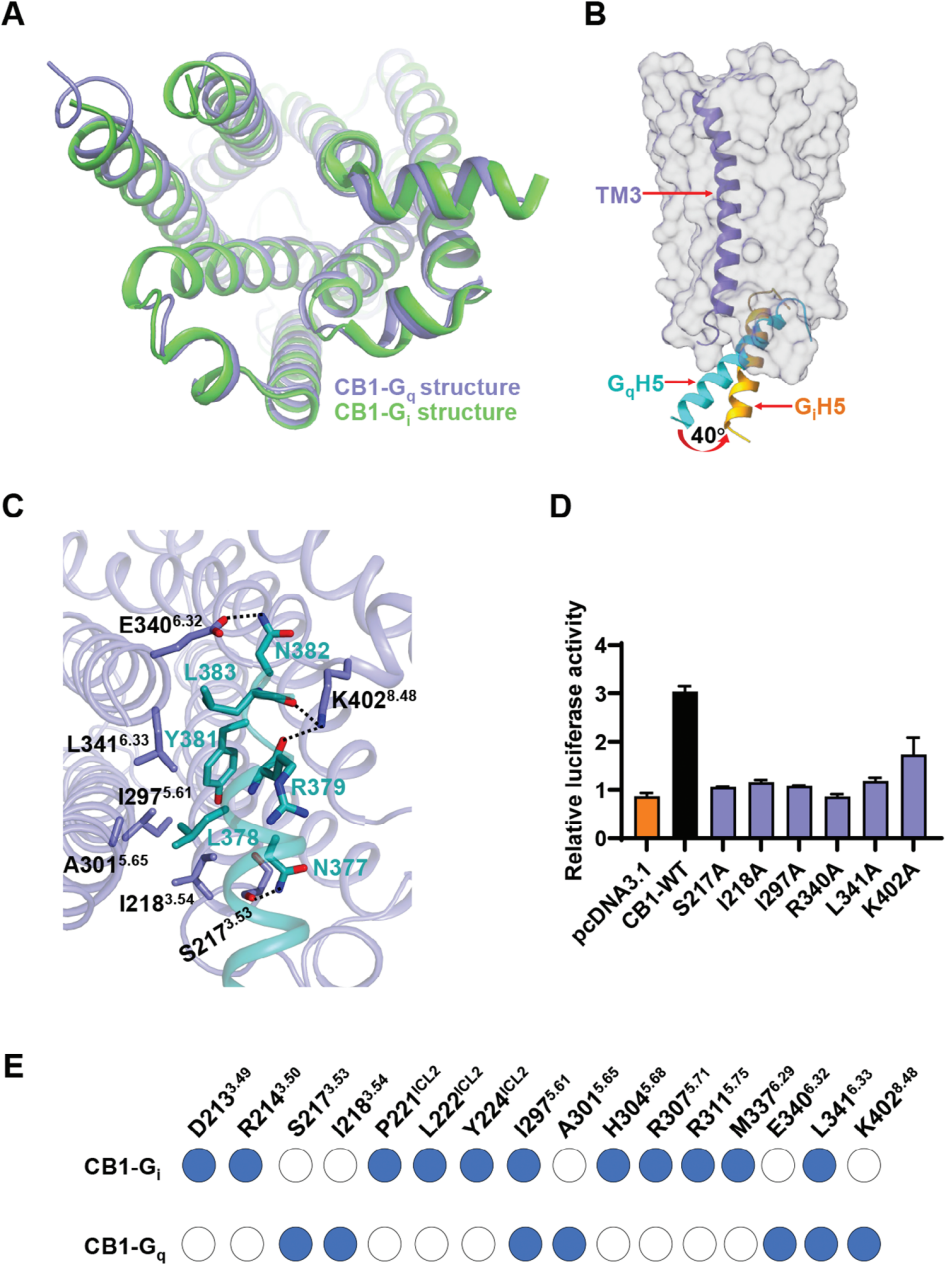
The G_q_ coupling feature to CB1. A,B) Structural comparison of the active CB1 receptors between the fenofibrate‐CB1‐miniG_s/q_ and the AM841‐CB1‐G_i_ complexes (A) and of α5‐ helix (H5) between G_q_ and G_i_ proteins (B). C) Detailed interactions of CB1 with the α5‐helix (cyan) of G_q_ in the fenofibrate‐CB1‐miniG_s/q_ structure. Polar interactions are highlighted as black dashed lines. D) Mutational effects of CB1's G_q_ coupling interface on fenofibrate (5 µM) induced increased calcium activities. Data are shown as the mean ± s.e.m. from three independent measurements. E) Comparison of CB1's residues that contact the corresponding downstream G protein subtypes, the G_i_ or G_q_, respectively.

Owing to the lack of the experimental structure of CB1‐G_q_ complex, the detailed interaction mechanism between CB1 and G_q_ is still ambiguous. The structure of CB1‐miniG_s/q_ complex uncovered that the interaction between miniG_s/q_ and CB1's cytoplasmic cavity was mainly contributed by the transmembrane helices TM3, TM5, TM6, and TM7 in CB1. The CB1‐G_q_ complex exhibited a global structure resembling other G_q_‐bound receptors, although a noticeable deviation in the α5‐helix distinguished it from the G_q_ protein in the GPR139‐G_q_ complex^[^ ^25^ ^]^ (PDB code: 7VUH), resulting in an 18° relative rotation of the G_q_ protein (Figure S6, Supporting Information). In contrast, a comparison of the miniG_s/q_‐ and G_i_‐CB1 (PDB code: 6KPG) structures revealed no specific conformational differences between the receptors (**Figure** **4**A), which differed from the human glucagon receptor.^[^ ^26^ ^]^ However, the α5‐helix of the G_q_ protein displayed a 40° clockwise rotation compared to that of the G_i_ protein (Figure 4B). This disparity suggests that CB1 adopts a unique coupling mode with the G_q_ protein, attributed to closer contacts between the α5‐helix of G_q_ and TM3 of CB1 (Figure 4B). We propose that the ability of CB1 to accommodate the α5‐helix of G_q_ serves as a determinant of G_q_ coupling specificity.

The interaction between the G_q_ protein and CB1 involved both polar and hydrophobic interactions. Specifically, the residue K402^8.48^ established the hydrogen bonding interactions with the O atoms of R379 and L383 in the G_q_’s α5‐helix, while the residue N377 formed the hydrogen bonding interaction with the hydroxyl group of S217^3.53^. Additionally, the residue N382 from G_q_’s α5‐helix formed the polar interaction with E340^6.32^. Moreover, the hydrophobic side chains of L383, Y381, and L378 on G_q_ protein directed toward the hydrophobic pocket (I218^3.54^, I297^5.61^, A301^5.65^, and L341^6.33^) of CB1 (Figure 4C). Mutations of these residues impaired the calcium‐induced luciferase accumulation of CB1 (Figure 4D). To illustrate the key residues of CB1 for the determinants of G_q_ or G_i_ coupling specificity, we performed a sequence alignment of the CB1 positions bound by the α5‐helix of G_q_ and G_i_ protein (PDB code: 6KPG). We observed significant differences in the detailed interactions of CB1 with G_q_ or G_i_ proteins, except for the residues I297^5.61^ and L341^6.33^ (Figure 4E).

## 3 Conclusion

In summary, the cryo‐EM structure reported in our study reveals the molecular recognition mechanism behind the precise recognition of the commercial drug fenofibrate by CB1. Fenofibrate was consolidated in the orthosteric pocket through hydrogen bonding interaction with the residue S173^2.60^, as well as through hydrophobic interactions. This work will facilitate further research into the pharmacological effects of fenofibrate on the human body and promote the design of new highly selective drugs targeting CB1 by comparing the similarities and differences in the recognition mechanisms of endogenous ligands, synthetic cannabinoids, and commercial drugs by CB1.^[^ ^27^ ^]^ Furthermore, the determined structures of CB1‐G protein complexes in previous studies mainly focused on G_i_ pathway. We first determined the cyro‐EM structure of CB1‐G_q_ complex and elucidated the coupling mechanism between CB1 and G_q_ protein. CB1 adopts the different coupling barcodes to recognize G_q_ and G_i_ proteins, respectively. This structure provides new insight into G protein selectivity of CB1 activation and a structural basis for designing highly selective drugs of CB1.

## 4 Experimental Section

### Materials and General Procedures

Fenofibrate was purchased from InnoChem Science & Technology. The detergent Lauryl Maltose Neopentyl Glycerol (LMNG) and Synthetic drop‐in substitute for Digitonin glyco‐diosgenin (GDN) used for receptor solubilization were purchased from Anatrace. Cholesterol hemisuccinate (CHS) was purchased from Sigma–Aldrich. Components of CB1‐fenofibrate‐miniG_s/q_‐Nb35 complex are expressed separately and assembled in vitro. Protein purification was performed at AKTA avant system.

### Calcium‐Induced Luciferase Accumulation Assay

The HEK293T cells were plated in 24 well plates and transfected with pGL4.30[luc2P/NFAT‐RE/Hygro] vector and wild‐type CB1 vector or CB1‐mutant vector by using Lipofectamine 2000. After 24 h, the cells were washed with PBS and treated with different concentrations of fenofibrate for 12 h. Then, the cells were harvested and lysed with passive lysis buffer contained in a luciferase reporter assay kit (Promega). The luciferase activity was normalized by protein amounts of cell lysates. The protein concentration was determined using a bicinchoninic acid (BCA) protein assay kit. The data are processed by graphpad prism10 and shown as the mean ± s.e.m. from three independent measurements.

### Expression and Purification of CB1

The construct of CB1 was modified from previously reported.^[^ ^19^ ^]^ Human wild‐type CB1 with the truncations of the residues 1–70 and 426–472, and the mutations (T210I, E273K, T283V, and R340E) was subcloned into a modified pTT5 mammalian expression vector containing a haemagglutinin (HA) signal sequence, a FLAG tag, 10×His tag and a tobacco etch virus (TEV) protease cleavage site at N‐terminus, followed by a thermostabilized BRIL and a PreScission protease site before CB1 sequence. Additionally, a LgBiT sequence was fused to the C‐terminal of the modified CB1 for increasing complex stability.^[^ ^20^ ^]^ CB1 was expressed in HEK293F cells using FreeStyle 293 Expression system (Invitrogen), and purified as previously described^[^ ^19^ ^]^ with the addition of 50 µm fenofibrate. Briefly, the cell pellet was lysed by hypotonic buffer (10 mm HEPES pH 7.5, 10 mm MgCl_2_, 20 mm KCl) for 2 h at 4 °C and centrifuged at 35 000 rpm for 30 min. The precipitate was washed once again by using a hypotonic buffer. Then the crude membrane was washed by hypertonic buffer (10 mm HEPES pH 7.5, 10 mm MgCl_2_, 20 mm KCl, 1 m NaCl) three times, and resuspended in cryopreservation solution (the hypotonic buffer supplemented with 30% glycerol(v/v)). The membrane suspension was frozen by liquid nitrogen and stored at −80 °C for further use. After thawing on ice, 50 µm fenofibrate was added to the solution and incubated at 4 °C for 2 h. For the solubilization of the membrane, an equal volume of the detergent (100 mm HEPES pH 7.5, 200 mm NaCl, 1.5% LMNG, 0.3% CHS) was mixed with the membrane suspension and incubated at 4 °C for 3 h. The mixture was centrifuged at 35 000 rpm for 30 min and the supernatant was collected and incubated with TALON resin overnight. The resin was loaded onto a gravity column and washed with 15 column volume (CV) of the washing buffer 1 (25 mm HEPES pH 7.5, 100 mm NaCl, 10% Glycerol, 0.1% LMNG/0.02% CHS, 30 mm imidazole) and 15 CV of the washing buffer 2 (25 mm HEPES pH 7.5, 100 mm NaCl, 10% Glycerol, 0.03% LMNG/0.006% CHS, 50 mm imidazole). Then the resin was eluted with 4 CV of the elution buffer (25 mm HEPES pH 7.5, 100 mm NaCl, 10% Glycerol, 0.01% LMNG/0.002% CHS, 250 mm imidazole, 50 µm fenofibrate). Finally, the CB1‐LgBiT protein was concentrated to ≈1 mg mL^−1^ for complex assembly.

### Expression and Purification of miniG_s/q_ and Nb35

The miniG_s/q_ construct and purification procedure used in this study were modified from the previously described.^[^ ^25^ ^]^ The miniG_s/q_ gene was subcloned into pET14a vector and expressed in BL21(DE3) cells. The miniG_s/q_ purification was performed by using Ni‐FF resin and further purified by using Superdex 75 Increase 10/300 GL column (Cytiva). Finally, the purified miniG_s/q_ were concentrated to 16 mg mL^−1^ for use.

Human Gβ_1_ with the N‐terminal 6xHis tag and C‐terminal HiBiT, and human wild type Gγ_2_ with C68S mutation were subcloned into the pFastBac Dual vector. The baculovirus was generated according to Bac‐to‐Bac system, and infected insect sf9 cells to produce the biomass for purification. The infected cells were harvested after 48 h and lysed by hypotonic and ultrasonic. The Gβ_1_(HiBiT)γ_2_(C68S) dimer was purified by using immobilized metal ion affinity chromatography (IMAC) and cation exchange chromatography (Mono Q). The peak fractions were concentrated to 1 mg mL^−1^ for use.

The Nanobody 35 (Nb35) with a C‐terminal 6xHis tag was expressed and purified as previously described.^[^ ^22^ ^]^ Briefly, Nb35 was expressed in BL21(DE3) cells and purified by using nickel affinity chromatography, followed by size‐exclusion chromatography using a Superdex 75 increase 10/300 GL column, and finally concentrated to 1 mg mL^−1^.

### Assembly and Purification of CB1‐Fenofibrate‐miniG_s/q_‐Nb35 Complex

The purified CB1‐LgBiT, miniG_s/q_, Gβ_1_(HiBiT)γ_2_(C68S), and Nb35 were mixed in a molar ratio of 1:2:2:2 with the addition of 50 µm fenofibrate and incubated at 4 °Cfor overnight. The assembled complex was further purified in the buffer (20 mm HEPES pH 7.5, 100 mm NaCl, 0.00075% (w/v) LMNG, 0.00025% (w/v) CHS, 0.00025% (w/v) GDN, 100 µm TCEP, 50 µm fenofibrate) by Superdex 200 Increase 10/300 GL column (Cytiva). The fractions with the component molar ratio of 1:1:1:1 were collected and concentrated to 3 mg mL^−1^.

### Cryo‐EM Sample Preparation and Data Collection

Three microliters of sample was applied to glow‐discharged holey carbon grid (Quantifoil 200 mesh, R1.2/1.3), and vitrified in liquid ethane using Vitrobot Mark IV (Thermo Fisher Scientific). The grid was blotted for 3 s with blot force −1 under chamber conditions of 100% humidity and 4 °C. The cryo‐EM movie stacks were collected on a Titan Krios microscope operated at 300 kV equipped with Gatan K3 summit direct electron camera and a Gatan energy filter (slit set to 20 eV). Data were recorded at a nominal magnification of 105000 in counting mode, corresponding to a magnified pixel size of 0.416 Å. The total dose is ≈60 e^−^ Å^−2^, divided into 40 frames. Each point was exposed for 1.997s with the dose rate ≈20 e^−^ Å^−2^ s^−1^. The defocus range was set from −0.8 to −2.0 in SerialEM software to collect 3347 movies for 3D reconstitution.

### Cryo‐EM Data Processing

The movies were imported into RELION 4.0^[^ ^28^ ^]^ and beam‐induced motion correction was performed with *MotionCor2* ^[^ ^29^ ^]^ binning 2 to the physical pixel size of 0.832 Å. The corrected pictures were imported into CryoSPARC 3.2.0.^[^ ^30^ ^]^ Patch CTF estimated and filtered pictures with CTF<3.5 Å. The remaining 2753 pictures were subjected to blob picking with a size range from 110 to 160 Å. After particles were extracted and 2D was classified, obvious GPCR‐miniG_s/q_ complex 2D classes were chosen as templates for template picking. The resulting particles were extracted with box size of 320 Å and subjected to one round 2D classification. The unclear 2D classes were excluded from the further 3D classification. The remaining 956199 particles were first Ab‐initio reconstructed into five volume classes, and the particles from the best class were chosen to perform second round 3D classification. The final best volume was subjected to non‐uniform refinement and local refinement with a mask on receptor and G alpha ras domain in CryoSPARC.

### Model Building and Refinement

The initial template of CB1 was from CB1‐AM841‐Gi structure (PDB:6KPG), and downstream G protein and Nb35 were from GPR139‐JNJ63533054‐miniG_s/q_ structure (PDB:7VUH). Agonist coordinates and geometry restraints were generated using CCP4.acedrg.^[^ ^31^ ^]^ The models were mutated using PyMOL (http://www.pymol.org/pymol) and then were rigidly fit into the EM density map using UCSF Chimera X.^[^ ^32^ ^]^ After iterative model building between Coot^[^ ^33^ ^]^ and Phenix.real_space_refinement,^[^ ^34^ ^]^ a good‐looking pose was generated. Based on this pose, a composite map was generated by phenix.combine_focused_maps from global refine and local refine map alignments. The final model was subjected to refinement on the composite map using phenix.real_space_refine in Phenix. The model geometry was evaluated using Molprobity.^[^ ^35^ ^]^ The map resolutions of global refinement and local refinement were calculated with gold‐standard FSC_halfmap‐halfmap_ = 0.143 criteria.

### MD Simulations

The fenofibrate‐bound CB1‐miniG_s/q_ complex was used as model. The receptor and ligand were used for simulations. CHARMM‐GUI membrane builder^[^ ^36^ ^]^ was used to generate the system. CB1 was encapsulated with a bilayer of around 100 POPC in each leaf. The TIP3P water model was filled with a height of 12.5 Å on both sides of the membrane along the *z*‐axis. The box dimension is ≈90 Å X 90 Å X 90 Å. NaCl (0.15 m) was used to neutralize charges. The protein was parameterized by CHARMM36m force field,^[^ ^37^ ^]^ while a conserved disulfide bond between C257 and C264 was fixed. D163^2.50^ and D213^3.49^ were kept protonated to mimic proton transportation during the active state. The ligand fenofibrate was parameterized by Antechamber^[^ ^38^ ^]^ and transferred to CHARMM‐suited format in CHARMM‐GUI. Minimization, equilibrium, and production runs were conducted in GROMACS‐2023^[^ ^39^ ^]^ version. A reduced harmonic restraint strategy was imposed in minimization and equilibrium, which leverage weights down to zero. Minimized with a maximum of 5000 steps with the steepest descent until convergence on 1000 kJ mol^−1^ nm^−1^ maximum force tolerance. In the equilibrium process, 0.25 ns with v‐rescale thermostat and 1.625 ns with v‐rescale thermostat and c‐rescale barostat were performed. Production run was running for a total of 200 ns, a time step of 2 fs for integration and saving trajectory per 100 ps with Nose–Hoover thermostat and Parrinello–Rahman barostat. During all md integrators, LINCS algorithm was used to assign covalently bonded hydrogen. For non‐bonded interaction, a 10–12 Å switch range and 12 Å cutoff were assigned, and long‐range interaction was calculated by the Particle Mesh Ewald (PME) method.^[^ ^40^ ^]^

### MD Analysis

The full trajectory was concatenated by gmx.trjconv and gmx.trjcat. Mdtraj.rmsd^[^ ^41^ ^]^ and mdtraj.compute_distances were used to calculate RMSD and the distance between denoted atoms. GetContacts (https://getcontacts.github.io/) was used to calculate contact frequency.

## Conflict of Interest

The authors declare no conflict of interest.

## Author Contributions

T.W., W.T., Z.Z., R.Z., and Z.L. contributed equally to this work. J.W. organized the project. F.L., W.T., T.W., and X.G. guided all the structural analysis. F.L. designed all the mutants for functional analysis. T.W., B.L., Q.G., K.Z., H.L., and J.C. developed the CB1 constructs and optimized protein expression. T.W. performed protein expression, prepared samples for cryo‐EM grids, and collected the cryo‐EM data. W.T. performed cryo‐EM map calculation, model building, structure refinement, and MD simulations. Z.L., Z.Z., and R.Z. performed the functional assay. J.W. and F.L. wrote the manuscript. All the authors have read and commented on the manuscript.

## Supporting information

Supporting Information

## Data Availability

The structure model and associated cryo‐EM data have been deposited in the Protein Data Bank (PDB) with entry 8K8J and Electron Microscopy Data Bank (EMDB) with entry EMD‐36951 (composite map), EMD‐36952 (global refine map), EMD‐36953 (local refine map). The unaligned raw movies were also provided in the Electron Microscopy Public Image Archive (EMPIAR) with entry EMPIAR‐11642.
