# Supplementary material for: Fenofibrate Recognition and Gq Protein Coupling Mechanisms of the Human Cannabinoid Receptor CB1
Source: Adv Sci (Weinh). 2024 Jan 31;11(14):2306311. doi: 10.1002/advs.202306311 (PMC11005724; doi:10.1002/advs.202306311)
Supplement: Supplementary file 1 — Supporting Information [file ADVS-11-2306311-s001.pdf]

ADVANCED SCIENCE
Open Access

Supporting Information

for *Adv. Sci.*, DOI 10.1002/advs.202306311

Fenofibrate Recognition and G_q Protein Coupling Mechanisms of the Human Cannabinoid Receptor CB1

Tianxin Wang, Wenqin Tang, Ziyi Zhao, Ran Zhao, Zhenyu Lv, Xuzhen Guo, Quanchang Gu, Boxiang Liu, Haoyu Lv, Jiayan Chen, Kaiquan Zhang, Fahui Li and Jiangyun Wang**

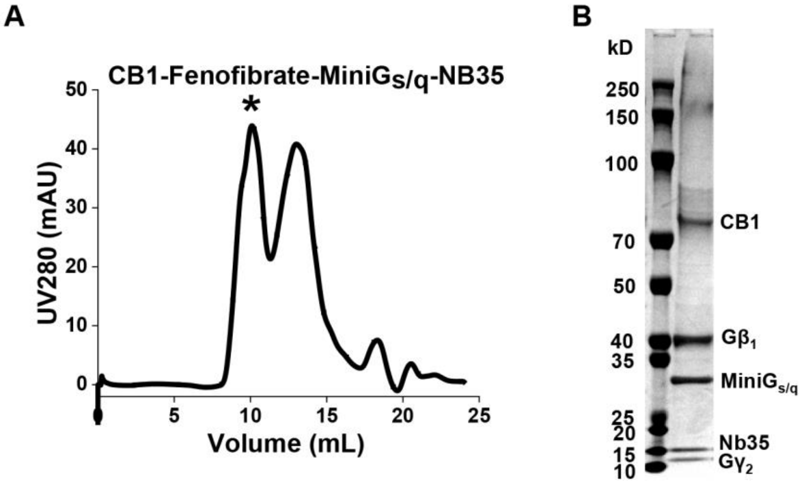

Figure S1. Purification of CB1-G_q-Nb35 complex. A) Representative size-exclusion chromatography elution profile of the purified CB1-G_q-Nb35 complex using Superdex200 Increase10/300GL. **B.** SDS-PAGE and Coomassie blue staining analysis of the size-exclusion chromatography peak of CB1-G_q-Nb35 complex.

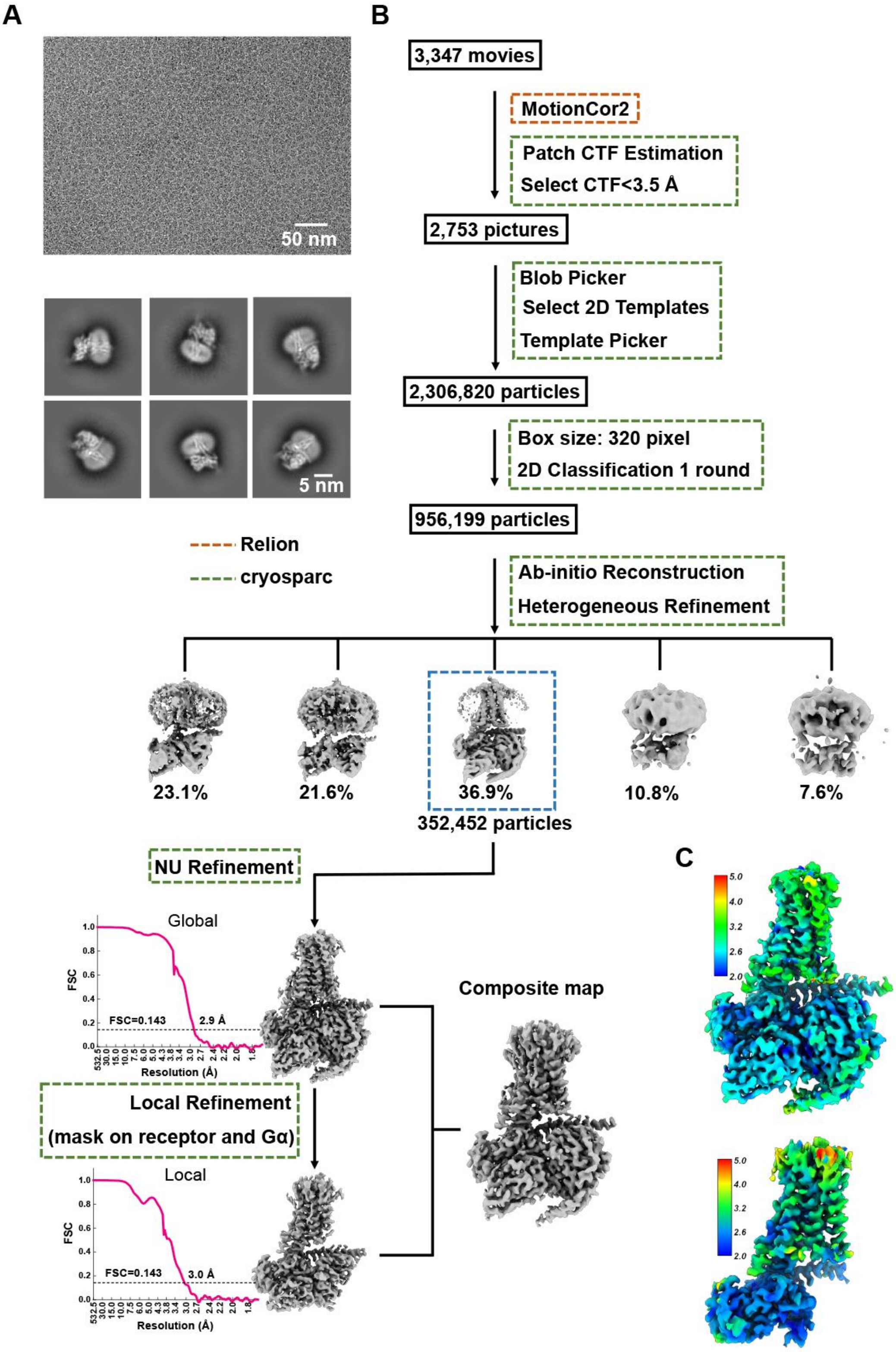

Figure S2. Cryo-EM processing and 3D reconstruction workflow. A) Representative cryo-EM image (upper panel) and 2D class averages (lower panel). B) Flow chart of cryo-EM data processing. C) 3D density map colored according to local resolution (Å) of the CB1-G_q-Nb35 complex.

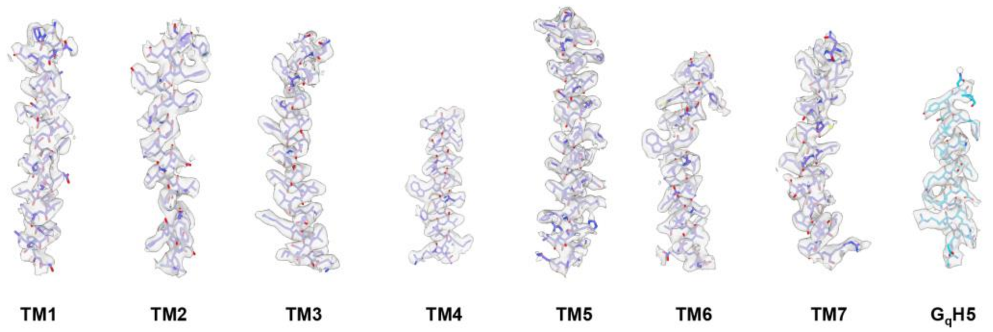

Figure S3. Cryo-EM density map of the Fenofibrate-CB1-G_q structure. Cryo-EM maps and models are shown for all transmembrane helices of the receptor and the α5-helix of G_q protein (G_qH5).

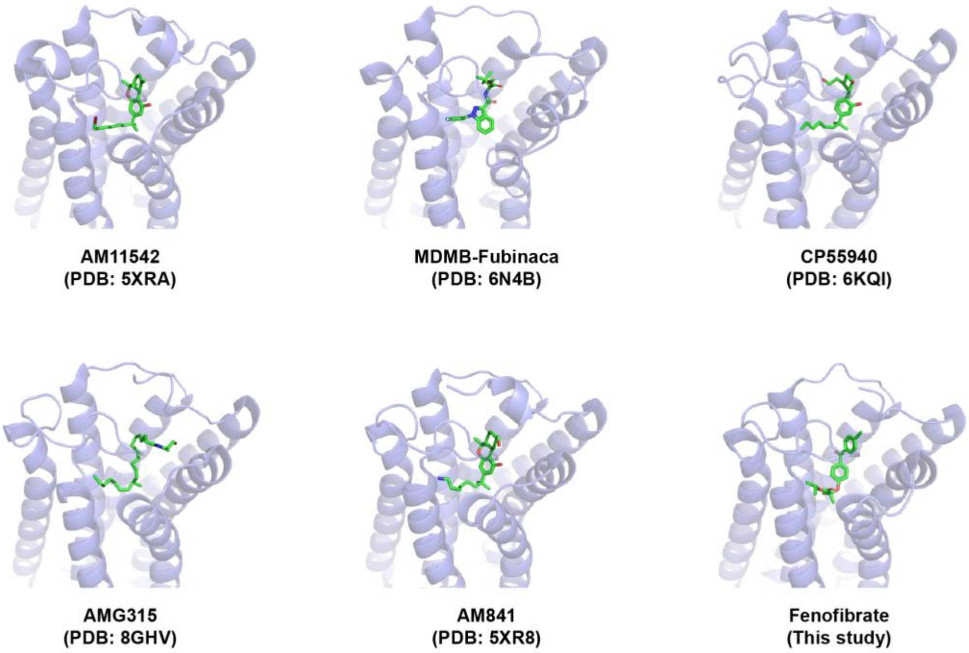

Figure S4. Comparison of the binding modes of the agonists-bound CB1 structures. The CB1 receptor and agonist were colored slate and green, respectively.

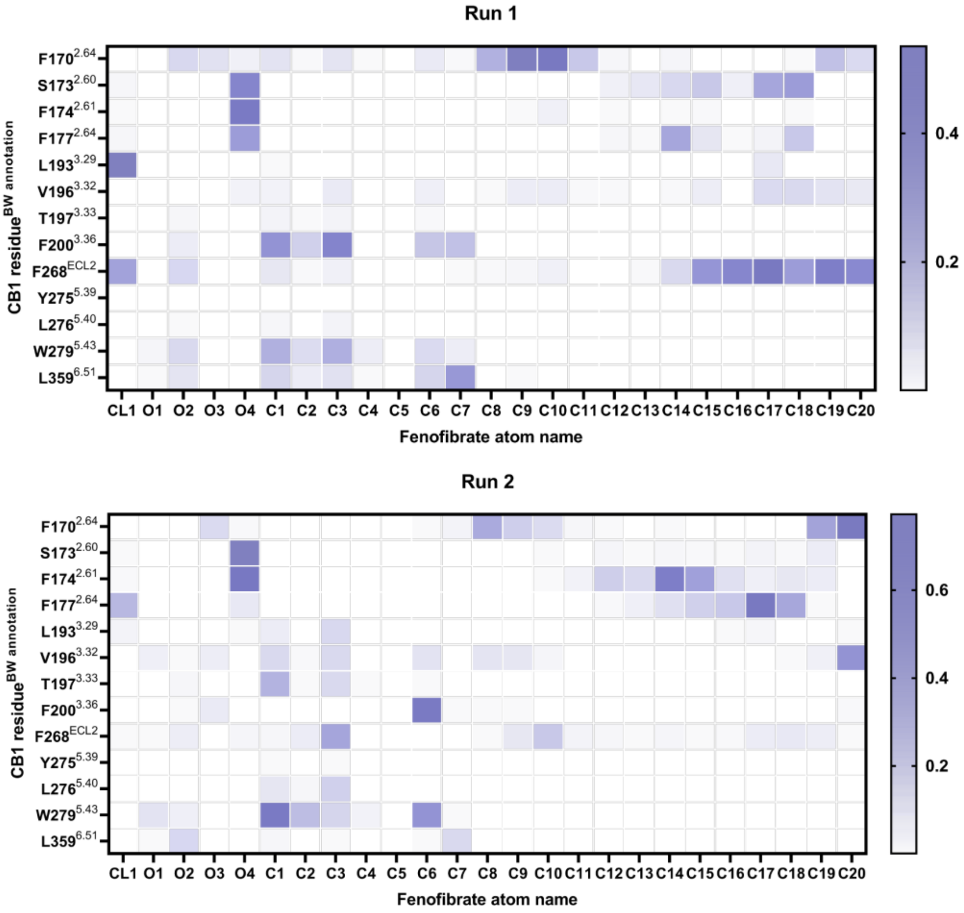

Figure S5. Molecular dynamics simulations of CB1 with Fenofibrate. Heatmap of contact frequencies of interaction between CB1-binding site residues and Fenofibrate atoms.

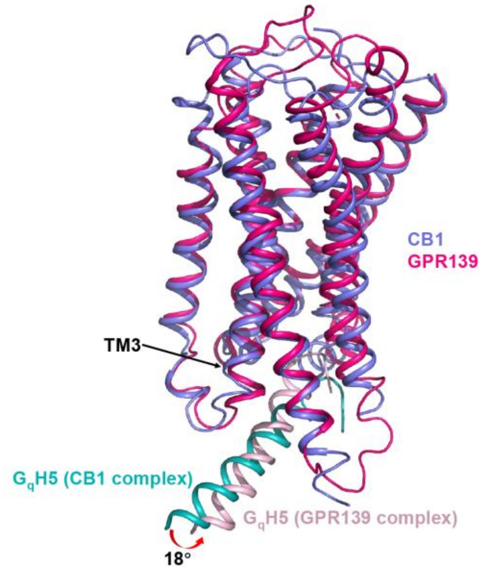

Figure S6. Structural comparison of the CB1-G_q and GPR139-G_q structures. The α5-helix of the G_q protein in CB1 structure takes a 18° relative rotation compared to that of G_q in GPR139 structure (PDB code: 7VUH).

Table S1. Cryo-EM data processing, model refinement and validation statistics.

**Data Collection**	**Global Refinement (Local Refinement)**
Voltage (kV)	300
Magnification	105,000
Total dose ($e^-/Å^2$)	60
Nominal defocus range (μm)	-0.8~-2.0
Physical pixel size (Å)	0.832
Micrographs collected	3,347
**Data Processing**	
Initial particles images (no.)	2,306,820
Final particles images (no.)	281,146
Map resolution (Å)	2.9 (3.0)
FSC threshold	0.143
Map sharpening B factor ($Å^2$)	128.5 (116.1)
Map resolution range (Å)	1.80-4.76 (1.79-6.37)
**Model refinement**	
Initial models used (PDB code)	6KPG and 7VUH
Map composition	
Non-hydrogen atoms	8,155
Protein residues	1,031
Ligand	1
R.m.s. deviations	
Bond lengths (Å)	0.004
Bond angles (Å)	0.549
Validation	
Molprobity score	1.83
Clashscore	8.91
Poor rotamers (%)	0.2
Ramachandran plot	
Favored (%)	94.89
Allowed (%)	5.11
Outliers (%)	0
